# The Combined Hypoglycemic Effect of Quercetagetin and Lutein from Marigold and Related Molecular Mechanisms in Mice

**DOI:** 10.3390/foods14244279

**Published:** 2025-12-12

**Authors:** Rongrong Wang, Chao Dang, Zhe Gao, Di Wu, Yunhe Lian, Xianghong Wang, Si Mi

**Affiliations:** 1 College of Food Science and Technology, Hebei Agricultural University, No. 2596 Lekai South Road, Baoding 071000, China; 2 Chenguang Biotech Group Co., Ltd., Handan 057250, China

**Keywords:** marigold, quercetagetin, lutein, hypoglycemic activity, molecular mechanism, gut microbiota

## Abstract

Marigold (*Tagetes erecta* L.) is rich in bioactive compounds, with lutein and quercetagetin as the primary components. However, the effects of these two substances on type 2 diabetes mellitus (T2DM) and their underlying molecular mechanisms remain incompletely understood. This study was designed to explore the hypoglycemic potential of quercetagetin and lutein, both individually and in combination, and to decipher the underlying molecular pathways. A T2DM mouse model was established using a high-fat diet (HFD) in combination with streptozotocin (STZ) administration. The results showed that quercetagetin and lutein effectively reduced fasting blood glucose and insulin levels, restored glucose metabolic homeostasis, and improved insulin sensitivity in T2DM mice. Additionally, these compounds improved blood lipid profiles, reduced the production of inflammatory factors, alleviated histological damage, and restored intestinal barrier function. Further mechanistic analysis revealed that quercetagetin and lutein could ameliorate intestinal dysbiosis, decrease intestinal lipopolysaccharide (LPS) content, mitigate local intestinal inflammation, and upregulate the expression of tight junction proteins. These alterations suggest that quercetagetin and lutein collectively contribute to the improvement of intestinal barrier dysfunction and systemic inflammation in type 2 diabetic (T2DM) mice.

## 1. Introduction

Type 2 diabetes mellitus (T2DM) is a metabolic disorder characterized by chronic low-grade inflammation, with a steadily increasing global prevalence [1]. According to the International Diabetes Federation, by 2045, over 194.5 million people worldwide could be affected by diabetes, with T2DM being the leading cause of this burden [2]. This condition is hallmarked by impaired metabolism of lipids, proteins, and carbohydrates, which contributes to the development of insulin resistance and impaired insulin secretion [3]. Currently, biguanides and sulfonylureas are among the therapeutic agents commonly used in the management of diabetes. However, these pharmaceuticals may exert off-target effects on organs like the heart, gastrointestinal tract, and liver, and have been linked to adverse outcomes including cardiovascular diseases, hepatic injury, and lactic acidosis [4]. Consequently, the exploration of natural hypoglycemic extracts with a favorable safety profile (i.e., fewer side effects) is of great clinical and translational significance. Previous studies have confirmed that bioactive substances such as alkaloids, flavonoids, phenolic acids, and glycosides exhibit hypoglycemic properties [5,6]. Currently, an increasing number of natural products are being investigated for their potential in treating T2DM and its comorbidities, either as standalone therapeutic interventions or in combination therapeutic regimens.

As the largest and most complex microecosystem in the human gut, the gut microbiota plays a crucial role in regulating both the normal physiological functions of the host and the initiation and progression of diseases [7]. Research has shown that a high-sugar and high-fat diet can induce gut microbiota dysbiosis [8], which is characterized by a marked increase in harmful bacteria and a decrease in beneficial bacteria in diabetic patients [9]. Moreover, disruption of the gut microbiota impairs the biological barrier of the intestine. Prolonged gut microbiota dysbiosis can trigger intestinal inflammation, leading to elevated circulating endotoxin levels, subsequently aggravated systemic inflammation, and ultimately the development of T2DM [10]. Therefore, targeting the impairment of intestinal barrier function induced by prolonged HFD intake has emerged as a key therapeutic strategy for T2DM.

*Tagetes erecta* L., a member of the *Asteraceae* family, exhibits multiple biological activities, including anti-inflammatory, antioxidant, and lipid-lowering properties. Studies have demonstrated that lutein, a bioactive component of *Tagetes erecta* L., can prevent diabetic complications and the development of cataracts [11]. In addition to lutein, the major flavonoids in *Tagetes erecta* L. are quercetagetin and quercetin. Previous laboratory studies have confirmed that quercetin exerts a hypoglycemic effect [6]. Structurally, quercetagetin possesses an additional hydroxyl group (-OH), which may enhance its biological activity. Wang [12] pointed out that quercetagetin shows strong in vitro antioxidant, anti-diabetic, and lipid-lowering activities, while its specific molecular mechanisms are not yet fully elucidated. In summary, the effects of quercetagetin and lutein on glucose-lipid metabolism, inflammatory responses, and gut microbiota in T2DM require further investigation.

In this study, a T2DM mouse model was established using a high-fat diet (HFD) combined with streptozotocin (STZ) administration. Using 16S rRNA sequencing, we investigated the key mediating roles and molecular mechanisms of quercetagetin and lutein in regulating glucose-lipid metabolism and intestinal barrier function.

## 2. Materials and Methods

### 2.1. Chemicals, Standards and Assay Kits

Quercetagetin standard (purity > 95%) was supplied by Chenguang Biotechnology Group Co., Ltd. (Handan, China). HPLC-grade lutein standard (purity > 98%) was purchased from McLean Biochemical Technology Co., Ltd. (Shanghai, China). Assay kits for determining total cholesterol, triglycerides, high-density lipoprotein cholesterol, low-density lipoprotein cholesterol, liver glycogen, alkaline phosphatase, glutamic oxaloacetic transaminase, glutamic pyruvic transaminase, and BCA protein were obtained from Jiancheng Bioengineering Institute (Nanjing, China). ELISA kits for insulin, glycated serum protein, lipopolysaccharide (LPS), tumor necrosis factor-α (TNF-α), interleukin-6 (IL-6), and interleukin-1β (IL-1β) were provided by Biotopped Technology Co., Ltd. (Beijing, China). Claudin 1 (1:1000, CAS#ab307692, 19 kDa) and Occludin (1:1000, CAS#ab216327, 59 kDa) were purchased from Abcam (Cambridge, UK). GAPDH (1:15,000, CAS#10494-1-AP, 36 kDa) and HRP-conjugated Affinipure Goat Anti-Rabbit IgG (1:15,000, CAS#00001-1) were obtained from Proteintech Group, Inc. (Wuhan, China).

### 2.2. Animals, Experimental Flow and Grouping

All animal experiments were approved by the Institutional Animal Care and Use Committee of Hebei Agricultural University (Approval No. 2022126). Eighty-four 6-week-old male KM mice (average weight 18 ± 2 g; License No.: SCXK (JING) 019-0010) were purchased from Spafford Biotechnology Ltd. (Beijing, China). All mice were maintained in a well-ventilated environment with an appropriate temperature (22 ± 2 °C) and relative humidity (55% ± 10%), under a 12 h light/12 h dark cycle.

After a 1-week acclimation period, 12 mice were randomly assigned to the control group (CK) and fed a normal standard diet. The remaining 72 mice were fed a commercial high-fat diet (HFD). The diet provided 60% of calories from fat (primarily lard), 20% from protein, and 20% from carbohydrate. Four weeks later, all mice were fasted for 12 h. Mice in the HFD group were intraperitoneally injected with streptozotocin (STZ) solution (0.1 mol/L in citrate buffer, pH 4.4) at a dose of 110 mg/kg body weight, while mice in the CK group received an equivalent volume of citrate buffer (pH 4.4). Fasting blood glucose (FBG) levels were measured at 3 and 7 days after STZ injection. Mice with FBG ≥ 11.1 mmol/L were considered successful T2DM models and included in subsequent experiments.

**Figure 1:**
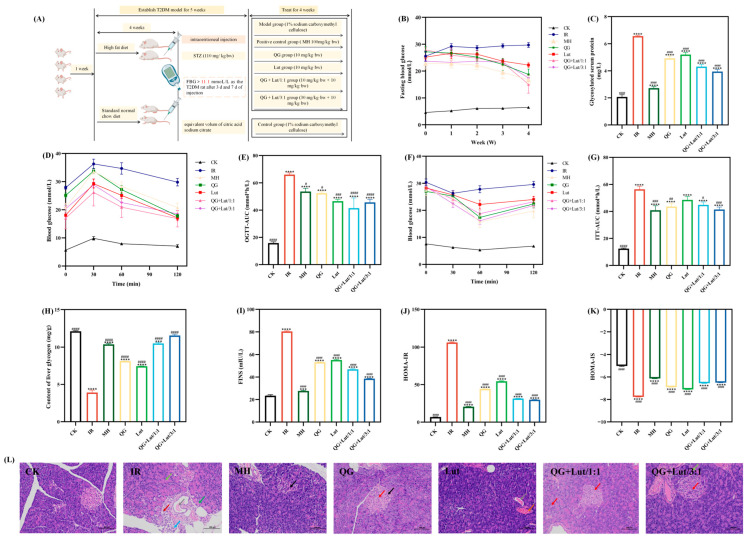
(**A**) Animal grouping and treatment, the effect of quercetagetin, lutein, and their combination on the (**B**) glucose content in the blood, (**C**) glycosylated serum protein content in serum, (**D**) blood glucose content recorded after glucose-administration for 0, 30, 60, 90, and 120 min, (**E**) area under the curve of oral glucose tolerance test (AUC-OGTT), (**F**) blood glucose content recorded after insulin-administration for 0, 30, 60, 90, and 120 min, (**G**) area under the curve of oral glucose tolerance test (AUC-ITT), (**H**) glycogen content in liver, (**I**) fasting insulin content in serum, (**J**) the insulin sensitivity index in serum, (**K**) the homeostasis model assessment-insulin resistance in serum, and (**L**) histopathological changes in pancreas (200× magnification). Red arrow: hydropic degeneration of islet cells and acinar cells; black arrow: pyknosis of islet cell nuclei; Light green arrow: increased zymogen granules; Green arrow: vacuolar degeneration of acinar cells; Gray arrow: eosinophilic material in ducts; blue arrow: granulocyte infiltration; dark red arrow: proliferation of periductal connective tissue. CK, blank control; IR, type 2 diabetic group; MH, metformin hydrochloride; QG, quercetagetin; Lut, lutein; QG + Lut/1:1, quercetagetin and lutein at a ratio of 1:1; QG + Lut/3:1, quercetagetin and lutein at a ratio of 3:1. Compared to the control group, *** *p* < 0.001 and **** *p* < 0.0001. Compared to the IR group, ^#^ *p* < 0.05, ^##^ *p* < 0.01, ^###^ *p* < 0.001, and ^####^ *p* < 0.0001 (*n* = 3).

The model mice were randomly divided into six groups: type 2 diabetic (IR) group (fed with HFD + 1% Na-CMC solution at 20 mg/kg body weight, *n* = 12), metformin hydrochloride (MH) group (fed with HFD + MH at 100 mg/kg body weight), quercetagetin (QG) group (fed with HFD + quercetagetin at 10 mg/kg body weight), lutein (Lut) group (HFD + lutein at 10 mg/kg body weight), quercetagetin and lutein (QG + Lut/1:1) group (HFD + quercetagetin 10 mg/kg + lutein 10 mg/kg), and quercetagetin and lutein (QG + Lut/3:1) group (HFD + quercetagetin 30 mg/kg + lutein 10 mg/kg). All compounds were suspended in 1% (*w*/*v*) sodium carboxymethyl cellulose (Na-CMC) solution by sonication. All groups received the same volume of vehicle (10 mL/kg body weight) via oral gavage once daily for 4 weeks [13]. During the experiment, food and water were provided ad libitum. Mice were observed daily for growth and health status; body weight, food intake, and water intake were recorded weekly, and the gavage volume was adjusted according to body weight. The specific animal experiment design is shown in Figure 1A. During outcome assessments (including histological scoring and Western blot band quantification), investigators were blinded to group allocation to minimize bias.

### 2.3. Sample Collection and Preparation

At the end of the intervention period, mice were fasted for 12 h (without food or water). Blood samples were collected via cardiac puncture after eyeball extraction, allowed to stand at room temperature for 30 min, and then centrifuged at 4 °C and 3000 rpm for 10 min. The separated serum was stored at −80 °C for subsequent analysis. After blood collection, mice were deeply anesthetized and euthanized by cervical dislocation. The liver, pancreas, and intestinal tissues were quickly excised, rinsed with pre-cooled 0.9% physiological saline, and blotted dry with filter paper. The pancreas and ileum tissues were each divided into two portions. One portion was placed in 10% neutral buffered formalin fixative for subsequent hematoxylin–eosin (HE) staining analysis.

### 2.4. Determination of Fasting Blood Glucose, Oral Glucose and Insulin Tolerance

On days 0, 7, 14, 21, and 28 of the intervention, FBG levels were measured from the tail vein using a Sinocare blood glucose meter (Changsha, China) after 12 h of fasting.

On day 23 of the intervention, all mice were fasted for 8 h. After measuring the fasting blood glucose level (0 min), mice were gavaged with a glucose solution at a dose of 2 g/kg body weight. Blood glucose levels were measured at 30, 60, and 120 min after gavage. The area under the oral glucose tolerance curve (AUC-OGTT) was calculated using the formula described by Fan et al. [14]:

AUC-OGTT = 0.5 × (BG 0 min + BG 30 min)/2 + 0.5 × (BG 30 min + BG 60 min)/2 + (BG 60 min + BG 120 min)/2

where BG represents blood glucose levels at 0, 30, 60, and 120 min after glucose gavage.

On day 26 of the intervention, all mice were fasted for 8 h. After measuring the fasting blood glucose level (0 min), insulin was intraperitoneally injected at a dose of 0.5 U/kg body weight. Blood glucose levels were measured at 30, 60, and 120 min after injection. The area under the insulin tolerance curve (AUC-ITT) was calculated using the same formula as AUC-OGTT, with BG representing blood glucose levels at the corresponding time points after insulin injection.

Serum insulin levels were determined using an ELISA kit (Biotopped Technology Co., Ltd., Beijing, China). The insulin sensitivity index (ISI) and insulin resistance index (HOMA-IR) were calculated using the formulas described by Xu et al. [15]:

ISI = ln [1/(FBG × FINS)]

HOMA-IR = (FBG × FINS)/22.5

### 2.5. Physiological Index Determination

The levels of glycated serum protein (GSP), tumor necrosis factor-α (TNF-α), interleukin-6 (IL-6), interleukin-1β (IL-1β), and lipopolysaccharide (LPS) were measured using assay kits from Biotopped Technology Co., Ltd. (Beijing, China) according to the manufacturer’s instructions.

The levels of total cholesterol (TC), triglycerides (TG), high-density lipoprotein cholesterol (HDL-C), low-density lipoprotein cholesterol (LDL-C), glutamic oxaloacetic transaminase (AST), glutamic pyruvic transaminase (ALT), and alkaline phosphatase (AKP) were determined using assay kits from Jiancheng Bioengineering Institute (Nanjing, China) following the manufacturer’s protocols.

### 2.6. Histopathological Analysis of Ileum and Pancreas Tissues

According to the method described by Liu et al. [13], the ileum and pancreas tissue sections were fixed in neutral formalin fixative (10%). The tissues were dehydrated using a gradient alcohol series (75–100%) and cleared by xylene for 1 h. Then the tissues were embedded in paraffin wax at 50–60 °C for 1.5 h. The embedding base mold was filled with melted paraffin wax, then the wax-impregnated tissue was placed into the embedding cassette, allowing the paraffin wax to solidify. Sections of approximately 4 μm thickness were cut from paraffin using a pathology sectioning machine, placed on 1% polylysine-treated slides, and baked for 2 h at 60 °C. Sections were stained with hematoxylin–eosin (HE) using standard protocols. Microscopic images were captured using an optical microscope (YS100, Nikon, Shanghai, China), and ileum histological scores were evaluated according to Table 1.

### 2.7. Western Blot Analysis of Ileal Tight Junction Proteins

Frozen mouse ileal tissues (100 mg) were thawed on ice and homogenized in 1 mL of RIPA lysis buffer (SEVEN, Beijing, China) containing 1% PMSF, protease, and phosphatase inhibitors (SEVEN, Beijing, China). The tissues were sufficiently ground and broken, and then placed on ice for 20 min for static lysis. Then, the tissues were centrifuged at 14,000× *g*/min for 20 min at 4 °C, and the supernatants were dispensed into centrifuge tubes. Determine the protein concentration of the supernatant by referring to the instructions of the BCA assay kit. The gel electrophoresis sample volume was calculated according to the protein sample volume of 30 μg. Protein samples were removed from −80 °C and protein samples were diluted and mixed with 5× protein pre-staining solution. Denaturation was performed in a metal bath at 100 °C for 5 min. Protein samples were cooled by running water and stored at −20 °C. Subsequently, proteins were separated using sodium dodecyl sulfate-polyacrylamide gel electrophoresis (SDS-PAGE), transferred to nitrocellulose (NC) membranes. Membranes were blocked with 5% skim milk powder, then incubated overnight at 4 °C with primary antibodies against Claudin 1, Occludin, and GAPDH. After washing, membranes were incubated with secondary antibodies for 1 h at room temperature. Protein bands were visualized using a highly sensitive chemiluminescence substrate (NCM Biotechnology, Suzhou, China) and a Junyi gel imaging system (JY04S-3E, Beijing, China). Band intensity was analyzed using Image J software (1.52v, National Institutes of Health, Bethesda, MD, USA).

### 2.8. Gut Microbiota Analysis by 16S rRNA Sequencing and Bioinformatics

Cecal feces were collected from the cecum of each experimental group of mice and analysis was performed referring to the method described by Qi et al. [16] with minor modifications. Cecal feces were collected from each group, and total DNA was extracted using a QIAamp-DNA Stool Mini Kit (QIAGEN, Beijing, China). The V3–V4 hypervariable region of the bacterial 16S rRNA gene was amplified using the universal primers 338F (5′-ACTCCTACGGGAGGCAGCAG-3′) and 806R (5′-GGACTACHVGGGTWTCTAAT-3′). PCR reactions were performed on an ABI GeneAmp^®^9700 PCR System (Applied Biosystems, Foster City, CA, USA) under the following conditions: initial denaturation at 95 °C for 3 min, followed by 27 cycles of denaturation at 95 °C for 30 s, annealing at 55 °C for 30 s, and extension at 72 °C for 45 s, with a final extension at 72 °C for 10 min. PCR products were quantified using a QuantiFluor^TM^-ST Handheld Fluorometer (Promega Corporation, Madison, WI, USA) and sequenced on an Illumina Miseq platform by Majorbio Bio-Pharm Technology Co., Ltd. (Shanghai, China). Raw sequencing reads were quality-filtered and analyzed using the Majorbio Cloud Platform (https://cloud.majorbio.com (accessed on 12 November 2024)). Each operational taxonomic unit (OTU) was categorized and the OTUs were evaluated using Alpha diversity (Simpson index and Bergerparker index), Beta diversity, and categorical composition analysis. LEfSe (version 1.1.x) software was used to analyze the significance of differences between groups. Alpha diversity indices (Simpson, Berger-Parker, Chao1, and Shannon indices) and Beta diversity (weighted/unweighted UniFrac distances) were calculated using QIIME2 (v2020.11). Statistical differences in alpha diversity between groups were assessed using the Kruskal–Wallis test, followed by Dunn’s post hoc test. Permutational multivariate analysis of variance (PERMANOVA) based on Bray–Curtis distances was used to evaluate beta diversity differences. Linear discriminant analysis effect size (LEfSe) was performed to identify differentially abundant taxa across groups (LDA score threshold > 3.0, *p* < 0.05).

### 2.9. Data Processing and Statistics

All results are presented as mean ± standard deviation (SD). All statistical analyses were performed using GraphPad Prism 8.0.2 (GraphPad Software Inc., San Diego, CA, USA) and SPSS 26.0 software. Normality and homogeneity of variance for all datasets were first assessed using the Shapiro–Wilk test and Levene’s test, respectively. For comparisons among multiple groups at a single time point, one-way analysis of variance (ANOVA) followed by Tukey’s post hoc test was applied when parametric assumptions were met; otherwise, the non-parametric Kruskal–Wallis test with Dunn’s post hoc test was used. For longitudinal data such as body weight, food/water intake, and oral glucose/insulin tolerance tests (OGTT/ITT), two-way repeated-measures ANOVA was employed, followed by Bonferroni correction for multiple comparisons. Beta-diversity analysis of gut microbiota was conducted using permutational multivariate analysis of variance (PERMANOVA) based on Bray–Curtis distances, while differential abundance of microbial taxa was identified by linear discriminant analysis effect size (LEfSe) with an LDA score threshold > 3.0 and *p* < 0.05. Relative protein expression levels from Western blotting were quantified using ImageJ 1.53t software (National Institutes of Health, USA). Statistical significance was set at *p* < 0.05. In figures, asterisks (*) indicate significant differences compared to the control (CK) group, and hash symbols (#) indicate significant differences compared to the diabetic model (IR) group. In tables, different lowercase letters denote statistically significant differences among treatment groups at the same time point (*p* < 0.05).

## 3. Results and Discussion

### 3.1. Dynamic Changes in Body Weight and Feed and Water Intake of Mice

Diabetic mice typically exhibit alterations in food intake, water intake, and body weight [17]. As shown in Table 2A, at week 0, the body weight of T2DM mice (all groups except CK) was lower than that of the CK group, consistent with the weight loss observed in hyperglycemic mice. The reason is that following high-dose streptozotocin (STZ) administration, insulin resistance may impair the digestive function and glucose utilization efficiency, ultimately resulting in weight loss [18]. As the intervention period progressed, the body weight of the model group gradually decreased compared with the control group (*p* < 0.05), exhibiting signs of emaciation. By contrast, the intervention groups showed varying degrees of weight gain, with the QG + Lut/3:1 group demonstrating the most significant improvement.

As presented in Table 2B,C, at week 0, T2DM mice exhibited higher food and water intake than the CK group. During the intervention, food and water intake increased in the CK group (likely due to weight gain), while the IR group sustained elevated intake—consistent with the polyphagia and polydipsia associated with diabetes. All intervention groups, however, exhibited reduced food and water intake, with the QG + Lut/1:1 and QG + Lut/3:1 groups showing the most marked improvements. These findings indicate that quercetagetin and lutein can alleviate polyphagia, polydipsia, and emaciation in T2DM mice.

### 3.2. Dynamic Changes in Blood Glucose and Insulin Levels of Mice

Persistent hyperglycemia is a major cause of diabetic complications, and fasting blood glucose (FBG) is a key indicator in the diagnosis and treatment of T2DM [19]. At the initiation of gavage, FBG levels of all groups except the CK group exceeded 11.1 mmol/L (Figure 1B), confirming successful establishment of the T2DM model. During the gavage period, FBG levels in the intervention groups showed a continuous downward trend. Furthermore, as the intervention proceeded to week 4, FBG levels in the QG + Lut/1:1 group and QG + Lut/3:1 groups were significantly lower than those in the IR group (*p* < 0.05).

Glycated serum protein (GSP) reflects blood glucose status over the past 1–3 weeks and is a sensitive indicator for evaluating glycemic control [20]. As shown in Figure 1C, GSP levels were significantly higher in the IR group than in the CK group (*p* < 0.0001), indicating poor glycemic control. All intervention groups exhibited significantly reduced GSP levels compared to the IR group (*p* < 0.0001), with the QG + Lut/3:1 group demonstrating the best efficacy.

Insulin resistance is a core feature of T2DM. Under normal physiological conditions, pancreatic β-cells enhance insulin secretion to compensate for decreased insulin sensitivity, thereby maintaining glucose tolerance. The oral glucose tolerance test (OGTT) and insulin tolerance test (ITT) are widely used to assess glucose tolerance and insulin sensitivity [21]. In the CK group, blood glucose levels gradually declined following glucose gavage and returned to normal by 120 min (Figure 1D). In contrast, the IR group sustained elevated blood glucose levels at 120 min, likely attributed to pancreatic islet cell damage and impaired function [22]. Oral administration with quercetagetin and lutein attenuated the elevation of blood glucose in T2DM mice, with the QG + Lut/1:1 group showing the most pronounced reduction. The IR group had a significantly higher OGTT area under the curve (AUC-OGTT) than the CK group (*p* < 0.05), while all intervention groups exhibited decreased AUC-OGTT values, with the QG + Lut/1:1 group exerting the optimal effect (Figure 1E).

As shown in Figure 1F, following insulin intraperitoneal injection, blood glucose value levels decreased significantly in all groups, and the IR group reached its lowest value at 30 min, while the intervention groups attained their lowest levels at 60 min post-injection, followed by a gradual increase in blood glucose. Compared to the IR group, the rate of blood glucose elevation was effectively suppressed in all intervention groups. The ITT area under the curve (AUC-ITT) was significantly higher in the IR group than in the control group (*p* < 0.05) (Figure 1G), but was significantly decreased in all intervention groups following treatment (*p* < 0.05).

The liver is a key target organ of insulin, and liver glycogen plays a crucial role in regulating insulin sensitivity [23]. As shown in Figure 1H, liver glycogen content was significantly lower in the IR group than in the CK group. All intervention groups exhibited increased liver glycogen levels, with the QG + Lut/3:1 group showing the most prominent effect. Chronic hyperglycemia can induce excessive insulin secretion, resulting in decreased insulin sensitivity and ultimately insulin resistance [24]. Compared to the control group, the IR group exhibited significantly higher insulin levels and HOMA-IR values (Figure 1I,J), both of which were decreased in all intervention groups, with the combined treatment groups showing the most notable reductions. Furthermore, the IR group had lower HOMA-IS values than the CK group (Figure 1K), and these values were increased following intervention—with the combined intervention groups demonstrating superior efficacy.

Furthermore, the combined treatment with a high-fat diet (HFD) and streptozotocin (STZ) induces the destruction of pancreatic β-cells [25]. Histopathological examination of pancreatic tissues via HE staining revealed that the CK group exhibited intact islet cells with regular arrangement and distinct borders (Figure 1L). In contrast, the IR group showed a decrease in the number and size of islet cells, disorganized arrangement, and was accompanied by inflammatory cell infiltration. All intervention groups exhibited attenuated pancreatic islet injury, with the QG + Lut/1:1 group showing the most pronounced improvement.

### 3.3. Variations in Serum Biochemical Indicators of Different Groups of Mice

**Figure 2:**
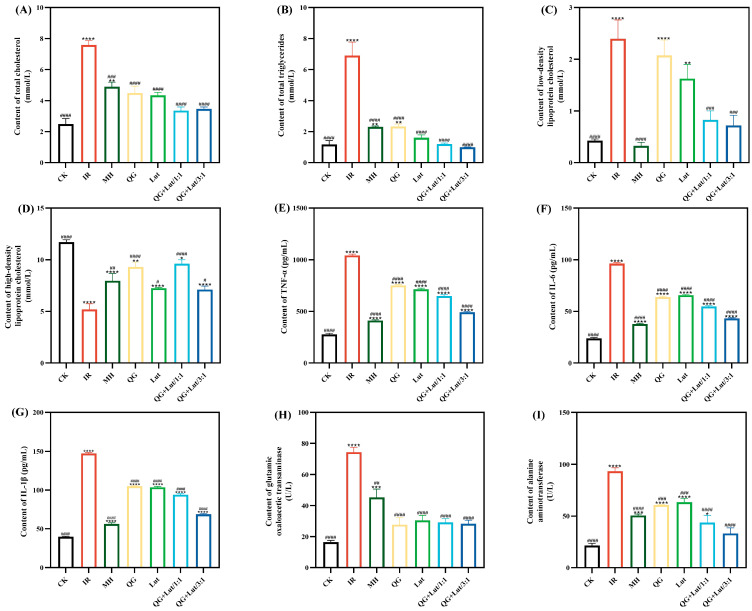
The effect of quercetagetin, lutein, and their combination on the content of (**A**) total cholesterol in serum, (**B**) triglycerides in serum, (**C**) low-density lipoprotein cholesterol in serum, (**D**) high-density lipoprotein cholesterol in serum, (**E**) tumor necrosis factor-α (TNF-α), (**F**) interleukin-6 (IL-6), (**G**) interleukin-1β (IL-1β), (**H**) glutamic oxaloacetic transaminase, and (**I**) alanine aminotransferase (*n* = 3). Compared to the control group, * *p* < 0.05, ** *p* < 0.01, *** *p* < 0.001 and **** *p* < 0.0001. Compared to the IR group, ^#^ *p* < 0.05, ^##^ *p* < 0.01, ^###^ *p* < 0.001, and ^####^ *p* < 0.0001 (*n* = 3).

Abnormal lipid metabolism is a common comorbidity of T2DM, characterized by elevated total cholesterol (TC), triglycerides (TG), and low-density lipoprotein cholesterol (LDL-C) levels—abnormalities that can contribute to organ damage and cardiovascular diseases [26]. Abnormalities of lipid metabolism are typically characterized by high levels of total cholesterol (TC), triglycerides (TG), low-density lipoprotein cholesterol (LDL-C), and low levels of high-density lipoprotein cholesterol (HDL-C) [27]. As shown in Figure 2A–D, the IR group exhibited significantly higher TC, TG, and LDL-C levels, and lower HDL-C levels compared to the CK group. Following 4 weeks of intervention, all treatment groups showed decreased TC, TG, and LDL-C levels, and increased HDL-C levels, with the QG + Lut/1:1 and QG + Lut/3:1 groups demonstrating significant improvements (*p* < 0.01).

Inflammatory responses contribute to the development of T2DM by inducing insulin resistance, which in turn elevates inflammatory cytokine levels, creating a vicious cycle that promotes disease progression and complications [28]. As shown in Figure 2E–G, the IR group exhibited significantly higher levels of TNF-α, IL-6, and IL-1β compared to the CK group. All intervention groups showed decreased levels of these inflammatory cytokines, indicating that quercetagetin and lutein can mitigate inflammatory responses in T2DM mice.

Aspartate transaminase (AST) and alanine transaminase (ALT) are key biomarkers of liver function [29]. Elevated serum AST and ALT levels in STZ-induced T2DM mice are likely due to hepatocellular injury induced by STZ. ALT is released into bloodstream upon hepatocellular damage, while AST is primarily localized in hepatic mitochondria and reflects the extent of liver necrosis [30]. Consistent with this, STZ-induced T2DM mice exhibited significantly higher AST and ALT levels than the CK group (*p* < 0.0001) (Figure 2H,I), which is indicative of hepatocellular injury. Following 28 days of intervention, AST and ALT levels were decreased in all treatment groups relative to the IR group, demonstrating a hepatoprotective effect of quercetagetin and lutein.

### 3.4. Changes in Intestinal Barrier Function of Different Groups of Mice

**Figure 3:**
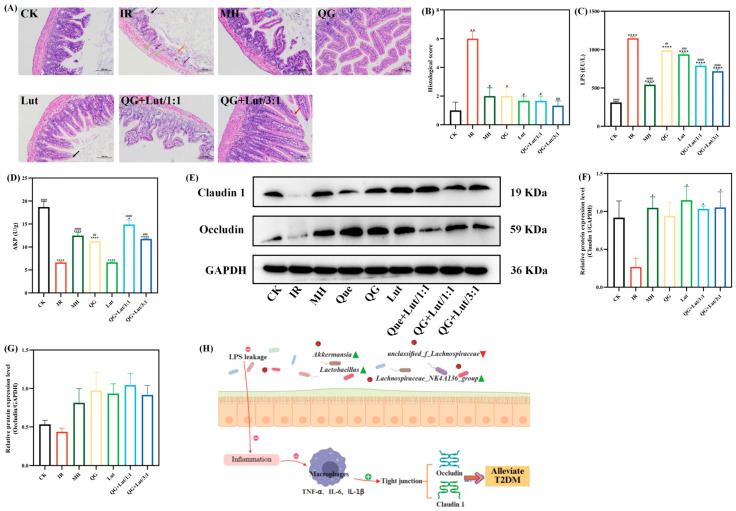
The effect of quercetagetin, lutein, and their combination on the (**A**) histopathological changes in ileum (200× magnification), Orange arrow: Intestinal tissue ulceration. Purple arrow: Lymphocyte infiltration. Black arrow: Necrosis and exfoliation of intestinal villus epithelial cells. Green arrow: Edema of intestinal gland epithelial cells Light green arrow: Infiltration of lymphocytes and macrophages. Blue arrow: Separation of intestinal villus epithelial cells from the lamina propria. Red arrow: Hydropic degeneration of intestinal villus epithelial cells. (**B**) histological score of ileum, (**C**) the content of lipopolysaccharide (LPS), (**D**) the content of alkaline phosphatase (AKP), (**E**) the immunoblot bands of the two target proteins, (**F**) the relative protein expression levels of Claudin1, (**G**) the relative protein expression levels of Occludin, and (**H**) Schematic diagram of the inflammatory mechanism (*n* = 3). Compared to the control group, * *p* < 0.05, ** *p* < 0.01, and **** *p* < 0.0001. Compared to the IR group, ^#^ *p* < 0.05, ^##^ *p* < 0.01, ^###^ *p* < 0.001, and ^####^ *p* < 0.0001 (*n* = 3).

Accumulating evidence suggests that T2DM is a chronic inflammatory disease closely linked to impaired intestinal homeostasis. In addition to the gut microbiota, the intestinal barrier plays a crucial role in maintaining intestinal homeostasis. Histopathological examination of ileal tissues revealed that the CK group had short intestinal villi composed of a single layer of columnar epithelium and goblet cells, abundant and densely arranged intestinal glands in the lamina propria, and a structurally clear myenteric layer with regularly arranged myoblasts (Figure 3A). The intestinal glands in the lamina propria were abundant, densely arranged, and short tubular, and there were few cup cells. The myenteric layer was clear in structure, and the myoblasts were arranged in a regular way, and no other obvious abnormalities were observed. In contrast to the CK group, the IR group showed multiple intestinal villi with apical autolysis and occasional vascular congestion, along with uneven thickness of the muscularis propria. Following intervention with quercetagetin and lutein, all treatment groups exhibited alleviated ileal lesions. Consistent with these findings, histological scoring yielded similar results (Figure 3B).

Lipopolysaccharide (LPS) is a component of the outer membrane of Gram-negative bacterial cell walls. It acts as a key inflammatory agonist and is regarded as a critical initiator of chronic low-grade inflammation [31]. As shown in Figure 3C, the IR group exhibited significantly higher LPS levels than the CK group (*p* < 0.0001). Following 28 d of intervention, LPS levels were reduced in all treatment groups, with the QG + Lut/3:1 group demonstrating the most pronounced reduction. Intestinal alkaline phosphatase (AKP) is a key enzyme involved in regulating intestinal microbiota and preserving epithelial integrity, and serves as a component of the intestinal chemical barrier [32]. The IR group had significantly lower AKP levels compared to the CK group (*p* < 0.0001), and these levels were significantly elevated in all intervention groups (Figure 3D).

Tight junction proteins (e.g., Claudin 1 and Occludin) are core structural proteins constituting the intestinal epithelial tight junction complex. Their upregulation directly enhances the physical connection strength between cells, forming a more stringent molecular barrier between the intestinal lumen and the systemic circulation [33]. Western blot analysis revealed that the IR group exhibited decreased relative expression of Claudin 1 and Occludin compared to the CK group (Figure 3E–G). Following intervention, the expression levels of these proteins were elevated in all treatment groups. The QG + Lut/1:1 group showed higher Occludin expression, while both QG + Lut/1:1 and QG + Lut/3:1 groups exhibited significantly higher Claudin 1 expression (*p* < 0.05). These findings suggest that quercetagetin and lutein can reduce intestinal permeability by upregulating the expression of tight junction proteins.

These results indicate that quercetagetin and lutein can prevent lipopolysaccharide (LPS) from penetrating intestinal epithelial cells, inhibit the inflammatory response, reduce the levels of pro-inflammatory cytokines, thereby upregulating the expression of tight junction proteins, decreasing intestinal permeability, and alleviating small intestinal inflammation, ultimately achieving the function of protecting the intestinal barrier. The mechanism diagram illustrating the protective effects of quercetagetin and lutein on the intestinal barrier is shown in Figure 3H.

### 3.5. Gut Microbiota Variations in Mice Exposed to Different Treatments

**Figure 4:**
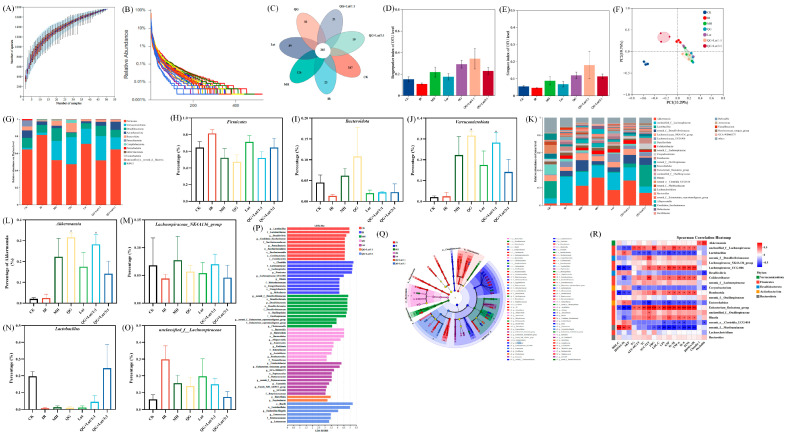
The effect of quercetagetin, lutein, and their combination on the gut microbiota structure of T2DM mice. (**A**) Species accumulation curves, (**B**) Rank-Abundance, (**C**) Venn diagram of OTUs, (**D**) Bergerparker index, (**E**) Simpson index, (**F**) β-diversity exhibited in the form of unweighted Unifrac PCoA diagram, (**G**) phylum level, relative abundance of (**H**) *Firmicutes*, (**I**) *Bacteroidetes*, (**J**) *Verrucomicrobia*, (**K**) genus level, relative abundance of (**L**) *Akkermansia*, (**M**) *Lachnospiraceae_NK4A136_group*, (**N**) *Lactobacillus*, (**O**) *f_Lachnospiraceae*, (**P**) linear discriminant analysis (LDA) histogram, (**Q**) cladograms, and (**R**) Superman’s correlation heatmap between the top 20 genus in relative abundance and T2DM-related biochemical indices (*n* = 3). Compared to the control group, * *p* < 0.05, ** *p* < 0.01, *** *p* < 0.001. Compared to the IR group, ^#^ *p* < 0.05 (*n* = 3).

Gut microbiota dysbiosis is closely linked to the development of T2DM [34]. 16S rRNA sequencing revealed that rarefaction curves gradually plateaued with increasing sequence numbers, indicating sufficient sequencing depth (Figure 4A,B). Venn diagram analysis showed 191 shared species across all groups, with 387 species unique to the CK group, 23 species unique to the IR group, 126 species unique to the MH group, 50 species unique to the QG group, 89 species unique to the Lut group, 21 species unique to the QG + Lut/1:1 group, and 19 species unique to the QG + Lut/3:1 group (Figure 4C), indicating that T2DM modulates intestinal flora diversity.

To assess the species diversity of the intestinal microbiota, we analyzed α-diversity indices, including the Simpson index and the Berger-Parker index. As shown in Figure 4D,E, both indices were slightly decreased in the IR group. Following intervention, these indices increased in all treatment groups, with the QG + Lut/1:1 exhibiting the most favorable outcomes. Principal coordinate analysis (PCoA) revealed distinct separation between the CK group and other groups, confirming that T2DM disrupts intestinal microbiota structure (Figure 4F). All intervention groups showed clear separation from the IR group, with the QG + Lut/1:1 group displaying an intestinal microbiota structure analogous to that of the CK group.

To investigate the compositional and abundance changes in microorganisms in the cecal contents, this study generated taxonomic bar plots at the phylum and genus levels by aligning sequencing data with reference databases. At the phylum level, the intestinal flora of experimental mice was mainly composed of *Firmicutes*, *Verrucomicrobia*, *Desulfobacterota, Actinobacteriota,* and *Bacteroidota* (Figure 4G). Compared to the CK group, the IR group had increased *Firmicutes* abundance and decreased *Bacteroidota* abundance (Figure 4H,I). After intervention with quercetagetin and lutein, the abundance of *Bacteroidota* showed an upward trend in all intervention groups, whereas the abundance of *Firmicutes* showed a downward trend. Studies have indicated that *Bacteroidota* can produce more carbohydrate-degrading enzymes compared with *Firmicutes* [35]. The treatment with quercetagetin and lutein inhibited *Firmicutes* while increasing the relative abundance of *Bacteroidota*, which may be attributed to the creation of a more favorable environment for *Bacteroidota*, but not for *Firmicutes*. Meanwhile, the abundance of *Verrucomicrobia* is strongly associated with gut health. *Verrucomicrobia* contributes to glucose homeostasis in the human gut and has anti-inflammatory properties that may further aid in intestinal health [36]. Quercetagetin and lutein increased *Verrucomicrobia* abundance compared to the IR group (Figure 4J). At the genus level, based on relative abundance, this study screened out 31 bacterial taxa with the most significant differences in abundance (Figure 4K), among which *Akkermansia*, *unclassified_f_Lachnospiraceae*, *Lachnospiraceae_NK4A136_group*, and *Lactobacillus* were the dominant genera. Further quantitative analysis was conducted on these 4 key genera. As shown in Figure 4L–O, compared with the IR group, the intervention with quercetagetin and lutein increased the abundances of *Akkermansia*, *Lachnospiraceae_NK4A136_group*, and *Lactobacillus*. According to the published reports, *Akkermansia* could reduce the blood insulin and total cholesterol concentrations, improve body insulin sensitivity, repair the intestinal barrier and maintain its integrity [37]. *Lactobacillus* had a positive role in anti-inflammation [38], whereas *Lachnospiraceae_NK4A136_group* possessed anti-inflammatory properties for intestinal mucosa repair [39]. Meanwhile, compared with the IR group, the intervention with quercetagetin and lutein decreased the abundances of *f_Lachnospiraceae* (Figure 4O). Previous studies have reported that obesity-related indicators were positively correlated with the abundance of *f_Lachnospiraceae* [40]. These results indicate that quercetagetin and lutein can improve the beneficial gut bacteria in diabetic mice at both the phylum and genus levels. In order to detect microbial species with significant abundance differences between different groups, the linear discriminant analysis effect size (LEfSe) method was performed (Figure 4P,Q). The representative bacteria of the CK group are *f_Lactobacillaceae* and *g_Lactobacillus*, while the representative bacteria of the IR group are *p_Firmicutes* and *g_Blautia*. Meanwhile, the results showed that *o_Lactobacillales* was associated with the effect of quercetagetin and lutein treatment in T2DM.

To explore potential relationships between microbial shifts and host phenotypes, Spearman’s correlation analysis was performed between the relative abundance of key genera and metabolic parameters (Figure 4R). *Lactobacillus* was significantly negatively correlated with pro-inflammatory cytokines (TNF-α, IL-6, and IL-1β), serum insulin, and lipid metabolism disorders; in contrast, *unclassified_f_Lachnospiraceae* exhibited the opposite correlation pattern. Meanwhile, *Akkermansia* showed a significant positive correlation with tight junction proteins (Claudin 1 and Occludin). These findings collectively confirm that quercetagetin and lutein can effectively ameliorate gut microbiota dysbiosis induced by hyperuricemia.

## 4. Conclusions

This study investigated the regulatory effects of quercetagetin and lutein on hyperglycemia, intestinal barrier dysfunction, and endocrine disorders in HFD/STZ-induced T2DM mice. The results showed that quercetagetin and lutein, either alone or in combination, reduced elevated fasting blood glucose and insulin levels, enhanced glucose and insulin tolerance, and alleviated insulin resistance. Additionally, these compounds repaired the intestinal barrier by mitigating inflammatory responses, reducing histological damage, and upregulating the expression of tight junction proteins (Claudin 1 and Occludin). Furthermore, quercetagetin and lutein ameliorated gut microbiota dysbiosis by restoring beneficial bacteria (*Akkermansia*, *Lachnospiraceae_NK4A136_group*, and *Lactobacillus*) and inhibiting harmful bacteria (*f_Lachnospiraceae*). These findings suggest that quercetagetin and lutein, as natural plant extracts, have great potential as functional food ingredients for the prevention and management of T2DM. However, the intervention doses employed in this study cannot be directly equated to recommended human intake levels, and translation of the research findings to clinical applications requires further validation through human trials.

## Figures and Tables

**Table 1:** Ileum histological score.

Lesion Type	Score	Standard
Inflammatory cell infiltration	0	None
	1	Mild
	2	Moderate
	3	Severe
Intestinal gland cell edema	0	None
	1	Mild
	2	Moderate
	3	Severe
Mucosal injury	0	None
	1	Mild
	2	Moderate
	3	Severe
Vascular congestion	0	None
	1	Mild
	2	Moderate
	3	Severe

**Table 2:** Effect of different dose levels of quercetagetin, lutein and their combinations on the body weight of mice during a 4-week administration. (**A**) Effect of different dose levels of quercetagetin, lutein and their combinations on the body weight of mice during a 4-week administration. (**B**) Effect of different dose levels of quercetagetin, lutein and their combinations on the feed intake of mice during a 4-week administration. (**C**) Effect of different dose levels of quercetagetin, lutein and their combinations on the water intake of mice during a 4-week administration.

(A)					
Groups (n = 10)	Body Weigh (g)				
	Week 0	Week 1	Week 2	Week 3	Week 4
CK	51.85 ± 0.84 ^a^	52.20 ± 2.40 ^b^	52.78 ± 2.25 ^a^	52.89 ± 3.71 ^a^	53.51 ± 5.31 ^a^
IR	48.05 ± 1.03 ^a^	46.57 ± 3.86 ^c^	46.71 ± 4.10 ^b^	46.21 ± 3.06 ^b^	45.12 ± 2.72 ^b^
MH	40.08 ± 5.79 ^b^	43.04 ± 4.07 ^c^	44.23 ± 4.43 ^b^	44.72 ± 4.78 ^b^	45.40 ± 3.71 ^b^
QG	49.64 ± 5.62 ^a^	47.88 ± 5.69 ^bc^	47.71 ± 6.76 ^ab^	48.77 ± 6.20 ^ab^	46.92 ± 6.80 ^b^
Lut	48.92 ± 3.25 ^a^	47.36 ± 3.12 ^bc^	47.23 ± 3.96 ^ab^	49.48 ± 1.15 ^ab^	49.53 ± 0.78 ^ab^
QG + Lut/1:1	49.26 ± 2.93 ^a^	46.65 ± 3.65 ^c^	47.44 ± 3.25 ^ab^	48.30 ± 3.51 ^ab^	49.18 ± 3.90 ^ab^
QG + Lut/3:1	47.08 ± 4.91 ^a^	47.53 ± 4.65 ^bc^	48.63 ± 4.38 ^ab^	49.64 ± 4.92 ^ab^	50.21 ± 4.57 ^ab^
(**B**)					
Groups (n = 10)	Feed intake (g)				
	Week 0	Week 1	Week 2	Week 3	Week 4
CK	4.72 ± 0.08 ^b^	4.76 ± 0.56 ^b^	5.25 ± 0.29 ^b^	5.40 ± 0.35 ^a^	5.64 ± 0.37 ^bc^
IR	6.35 ± 0.06 ^a^	6.50 ± 0.49 ^a^	6.86 ± 1.08 ^a^	7.06 ± 0.10 ^a^	7.34 ± 0.16 ^a^
MH	6.19 ± 0.13 ^a^	5.95 ± 0.19 ^a^	5.85 ± 0.70 ^ab^	5.47 ± 0.49 ^c^	5.28 ± 0.18 ^bcd^
QG	6.31 ± 0.08 ^a^	6.22 ± 0.03 ^a^	6.00 ± 0.11 ^ab^	5.46 ± 0.31 ^c^	4.65 ± 0.30 ^d^
Lut	6.29 ± 0.07 ^a^	6.20 ± 0.20 ^a^	6.11 ± 0.98 ^ab^	6.02 ± 0.58 ^bc^	5.42 ± 0.33 ^bcd^
QG + Lut/1:1	6.38 ± 0.42 ^a^	6.30 ± 0.06 ^a^	6.35 ± 0.73 ^ab^	5.44 ± 0.57 ^c^	5.02 ± 0.60 ^cd^
QG + Lut/3:1	6.23 ± 0.38 ^a^	6.21 ± 0.38 ^a^	6.14 ± 0.49 ^ab^	5.97 ± 0.68 ^bc^	5.57 ± 1.05 ^bc^
(**C**)					
Groups (n = 10)	Water intake (mL)				
	0	1	2	3	4
CK	7.24 ± 0.20 ^e^	7.37 ± 1.14 ^c^	7.63 ± 2.39 ^b^	7.70 ± 1.40 ^c^	8.07 ± 1.11 ^d^
IR	13.30 ± 0.42 ^abc^	14.13 ± 1.43 ^a^	14.43 ± 0.92 ^a^	15.59 ± 0.68 ^a^	15.60 ± 1.31 ^b^
MH	13.54 ± 0.36 ^a^	13.17 ± 0.30 ^ab^	12.65 ± 0.43 ^a^	12.32 ± 0.41 ^b^	12.17 ± 0.74 ^c^
QG	13.04 ± 0.08 ^cd^	12.64 ± 0.50 ^ab^	11.61 ± 2.30 ^a^	11.26 ± 0.53 ^b^	11.13 ± 0.35 ^c^
Lut	12.86 ± 0.15 ^d^	12.77 ± 0.73 ^ab^	12.17 ± 0.39 ^a^	11.44 ± 1.86 ^b^	10.38 ± 0.54 ^c^
QG + Lut/1:1	12.98 ± 0.19 ^cd^	12.38 ± 0.59 ^b^	11.53 ± 3.15 ^a^	11.34 ± 0.59 ^b^	11.14 ± 0.68 ^c^
QG + Lut/3:1	13.11 ± 0.12 ^bcd^	12.80 ± 0.34 ^ab^	11.49 ± 1.14 ^a^	11.41 ± 0.44 ^b^	11.40 ± 0.36 ^c^

***Note:*** Different lowercase letters represent significant differences between different groups at the same time. CK, blank control; IR, type 2 diabetic group; MH, metformin hydrochloride; QG, quercetagetin; Lut, lutein; QG + Lut/1:1, quercetagetin and lutein at a ratio of 1:1; QG + Lut/3:1, quercetagetin and lutein at a ratio of 3:1.

## Data Availability

The original contributions presented in the study are included in the article, further inquiries can be directed to the corresponding authors.
